# ERRATUM: Prevention of amphotericin B nephrotoxicity through use of phytotherapeutic medication

**DOI:** 10.1590/1980-220X-REEUSP-2024-ER10en

**Published:** 2024-09-23

In the article “Prevention of amphotericin B nephrotoxicity through use of phytotherapeutic medication”, with the DOI: https://doi.org/10.1590/S0080-623420150000700011, published by the journal: “Revista da Escola de Enfermagem da USP, volume 49(Esp) of 2015, p. 73–78, on page 78:

**Where it was written:**

## Financial support

Fundação de Amparo à Pesquisa do Estado de São Paulo – FAPESP. Projects 2011/24028-6 and 2013/26560-2.

**Now read:**

## Financial support

Fundação de Amparo à Pesquisa do Estado de São Paulo – FAPESP. Projects 2011/24028-6 and 2013/26560-2. This study was financed in part by the Coordenação de Aperfeiçoamento de Pessoal de Nível Superior – Brasil (CAPES) – Finance Code 001.

