# Supplementary material for: ERRATUM: Prevention of amphotericin B nephrotoxicity through use of phytotherapeutic medication
Source: Rev Esc Enferm USP. 2024 Sep 23;58:e2024ER10. doi: 10.1590/1980-220X-REEUSP-2024-ER10en (PMC11421568; doi:10.1590/1980-220X-REEUSP-2024-ER10en)
Supplement: Translation

# Translation: ERRATA: Prevenção da nefrotoxicidade da anfotericina B por meio do uso de fitomedicamentos

**DOI:** 10.1590/1980-220X-REEUSP-2024-ER10pt

No artigo “Prevenção da nefrotoxicidade da anfotericina B por meio do uso de fitomedicamentos”, com número de: DOI: https://doi.org/10.1590/S0080-623420150000700011, publicado na Revista da Escola de Enfermagem da USP, volume 49(Esp) de 2015, p. 74–79, na página 79:

**Onde se lia:**

## Apoio financeiro

Fundação de Amparo à Pesquisa do Estado de São Paulo – FAPESP. Projetos 2011/24028-6 e 2013/26560-2.

**Leia-se:**

## Apoio financeiro

Fundação de Amparo à Pesquisa do Estado de São Paulo – FAPESP. Projetos 2011/24028-6 e 2013/26560-2. O presente trabalho foi realizado com apoio da Coordenação e Aperfeiçoamento de Pessoal de Nível Superior – Brasil (CAPES) – Código de Financiamento 001.
